# A comparison of methods for the isolation and separation of extracellular vesicles from protein and lipid particles in human serum

**DOI:** 10.1038/s41598-020-57497-7

**Published:** 2020-01-23

**Authors:** K. Brennan, K. Martin, S. P. FitzGerald, J. O’Sullivan, Y. Wu, A. Blanco, C. Richardson, M. M. Mc Gee

**Affiliations:** 1 UCD School of Biomolecular & Biomedical Science, Conway Institute, University College Dublin (UCD), Belfield, Dublin, 4 Ireland; 2 Randox Teoranta, Meenmore, Dungloe, Donegal Ireland; 3 Randox Laboratories Ltd., Crumlin, Antrim United Kingdom; 4 Trinity Translational Medicine Institute (TTMI), Department of Surgery, Trinity College Dublin, St James’s Hospital, Dublin, Ireland; 5 UCD Conway Flow Cytometry Core, Conway Institute, University College Dublin (UCD), Dublin, Ireland

**Keywords:** Extracellular signalling molecules, Multivesicular bodies

## Abstract

Extracellular vesicles (EVs) are nano-sized vesicles containing nucleic acid and protein cargo that are released from a multitude of cell types and have gained significant interest as potential diagnostic biomarkers. Human serum is a rich source of readily accessible EVs; however, the separation of EVs from serum proteins and non-EV lipid particles represents a considerable challenge. In this study, we compared the most commonly used isolation techniques, either alone or in combination, for the isolation of EVs from 200 µl of human serum and their separation from non-EV protein and lipid particles present in serum. The size and yield of particles isolated by each method was determined by nanoparticle tracking analysis, with the variation in particle size distribution being used to determine the relative impact of lipoproteins and protein aggregates on the isolated EV population. Purification of EVs from soluble protein was determined by calculating the ratio of EV particle count to protein concentration. Finally, lipoprotein particles co-isolated with EVs was determined by Western blot analysis of lipoprotein markers APOB and APOE. Overall, this study reveals that the choice of EV isolation procedure significantly impacts EV yield from human serum, together with the presence of lipoprotein and protein contaminants.

## Introduction

Extracellular vesicles (EVs) were originally identified in reticulocytes as a means of disposing of obsolete membrane proteins such as α4β1 and transferrin receptor during reticulocyte maturation^1–3^, and have since been shown to participate in cell-cell signalling via transfer of proteins, nucleic acids and metabolites^4–6^. EVs have been identified in a diverse range of human biofluids including serum, plasma, urine, saliva, breast milk, amniotic fluid, ascites fluid, cerebrospinal fluid and bile^7,8^. These EVs are classified into three groups; exosomes, microvesicles and apoptotic bodies depending on their size, biogenesis and method of cellular release. Microvesicles and apoptotic bodies generally range from 100 to 1000 nm and 1–4 µm respectively, and are formed by budding from the plasma membrane^4,9^. In contrast, exosomes have a diameter of 30–150 nm and are formed by inward budding of the late endosome lumen to form a multivesicular body (MVB) that is secreted by fusion with the plasma membrane^10^. The overlap in exosome and microvesicle size (100–150 nm) and density (1.08–1.19 g/ml) makes it difficult to distinguish the two groups and as a result exosomes are often defined by their content of endosome-associated proteins including tetraspanins CD9, CD63, and CD81. However, since microvesicles from haematopoietic cells are also enriched for endosomal proteins such as CD63 and CD81^11^ exosomes and microvesicles <150 nm are collectively referred to as small extracellular vesicles (sEVs)^12^.

EV secretion has been shown to be elevated in response to inflammation^13^, hypoxia^14,15^ and an acidic microenvironment^16,17^ and is associated with human diseases such as cancer, where secretion levels have been shown to correlate with tumour invasiveness^18^. Tumour cells can manipulate their microenvironment by using EV-based cell-cell communication to induce immunosuppression via expansion of myeloid-derived suppressor cells^19,20^. Moreover, EV delivery of miRNA promotes angiogenesis in a variety of tumours^21–24^. The presence of miRNA and cancer associated proteins inside cancer-derived EVs has led to the identification of several potential disease biomarkers such as CPNE3 in colorectal cancer EVs^25^, miR-451a in non-small cell lung cancer EVs^26^, and miR-451a in pancreatic ductal adenocarcinoma EVs^27^, all of which are associated with poor disease-free survival.

The potential of EVs as minimally invasive liquid biopsies has accelerated proteomic and genomic EV profiling research for the identification of informative biomarkers for disease diagnosis, prognosis and longitudinal monitoring. However, while many EV profiling studies to date are based on EVs secreted from cells grown in culture, there is a need to validate potential biomarkers in physiologically relevant biofluids such as blood and saliva, or to profile these primary fluids directly. Serum and plasma are attractive sources of EV-based biomarkers as blood sample acquisition is a minimally invasive procedure and tumour cells release greater amounts of circulating EVs into the bloodstream^28,29^. Moreover, frozen biobanked serum is a valuable source of EVs for retrospective studies.

The exploitation of EVs as a biomarker source requires the use of methods for the isolation of EVs from small volumes of human serum, and separation from non-EV protein and lipoprotein that is abundant in human serum. The isolation and purification of EVs from serum is prone to several challenges related to high serum viscosity together with high abundance of serum proteins, such as albumin and globulins, and non-EV lipid particles such as chylomicrons and lipoprotein particles. These lipoprotein particles and soluble proteins can interfere with particle counts and biomarker analysis with HDL particles reported to contain miRNAs^30–32^. Furthermore, the level of chylomicrons and lipoprotein particles can vary greatly from individual to individual and is influenced by diet, genetics and race^33–38^ adding further complexity to biomarker validation studies. Therefore, separation of serum EVs from soluble proteins and non-EV lipid particles is critically important for the development of strategies for biomarker discovery and validation.

EVs can be distinguished from chylomicrons, VLDL, IDL, and LDL based on differences in density, with these particles having lower density than EVs (<1.063 g/mL). In contrast, HDLs have a density similar to EVs (1.06–1.21 g/mL) and therefore can only be separated from EVs based on differences in size (Table 1). A variety of methods are used to isolate EVs, each with their advantages and disadvantages. Ultracentrifugation uses centrifugal force to separate and purify EVs by using high centrifugal speed for sufficient time for individual EVs to travel the length of the tube into a pellet while being less efficient at pelleting smaller/less dense particles. Repeated centrifugation can reduce the amount of non-EV particles co-isolated with the EVs, however, it also results in reduced particle yield due to lost and damaged EVs. Polymer-based precipitation uses volume-excluding polymers to reduce the solubility of EVs and similarly sized proteins and particles, which are subsequently isolated using low speed centrifugation. However, polymer-based approaches require the use of protein removal kits to reduce the amount of coprecipitated proteins. Size exclusion chromatography (SEC) using the IZON qEV columns allows EVs larger than 70 nm to be separated from smaller particles and proteins that take longer to pass through the column, however this method does not completely separate EVs from non-EV material and the presence of EVs in multiple fractions leads to dilute samples that often need to be pooled and requires and additional concentration step. Density gradient centrifugation uses a density gradient medium such as iodixanol and centrifugal force to separate and purify EVs based on their buoyant density. A second ultracentrifugation step is then required to isolate EVs from a fixed density range which can result in the loss and damage of some EVs while co-isolating non-EV particles of a similar density. While several studies have compared some of these methods, studies relating to small volumes of serum or plasma have either compared different commercial EV isolation kits^39^, or compared commercial EV isolation kits to size exclusion columns or ultracentrifugation^40,41^ while density gradients have not been compared and are normally used for large volume EV isolations such as cell culture conditioned media^42^.

**Table 1:** Size and density ranges of the different chylomicrons, lipoproteins and EVs.

Particle type	Size (nm)	Density (g/ml)
Chylomicrons	75 to 1200	<0.95
chylomicron remnants	30–80	0.95–1.006
Very low-density lipoproteins (VLDL)	30–80	0.95–1.006
Intermediate-density lipoproteins (IDL)	23–27	1.006–1.019
Low-density lipoproteins (LDL)	18–23	1.019–1.063
High-density lipoproteins (HDL)	7–13	1.063–1.21
Extracellular vesicles (EVs)	30–1000	1.06–1.21

**Figure 1:**
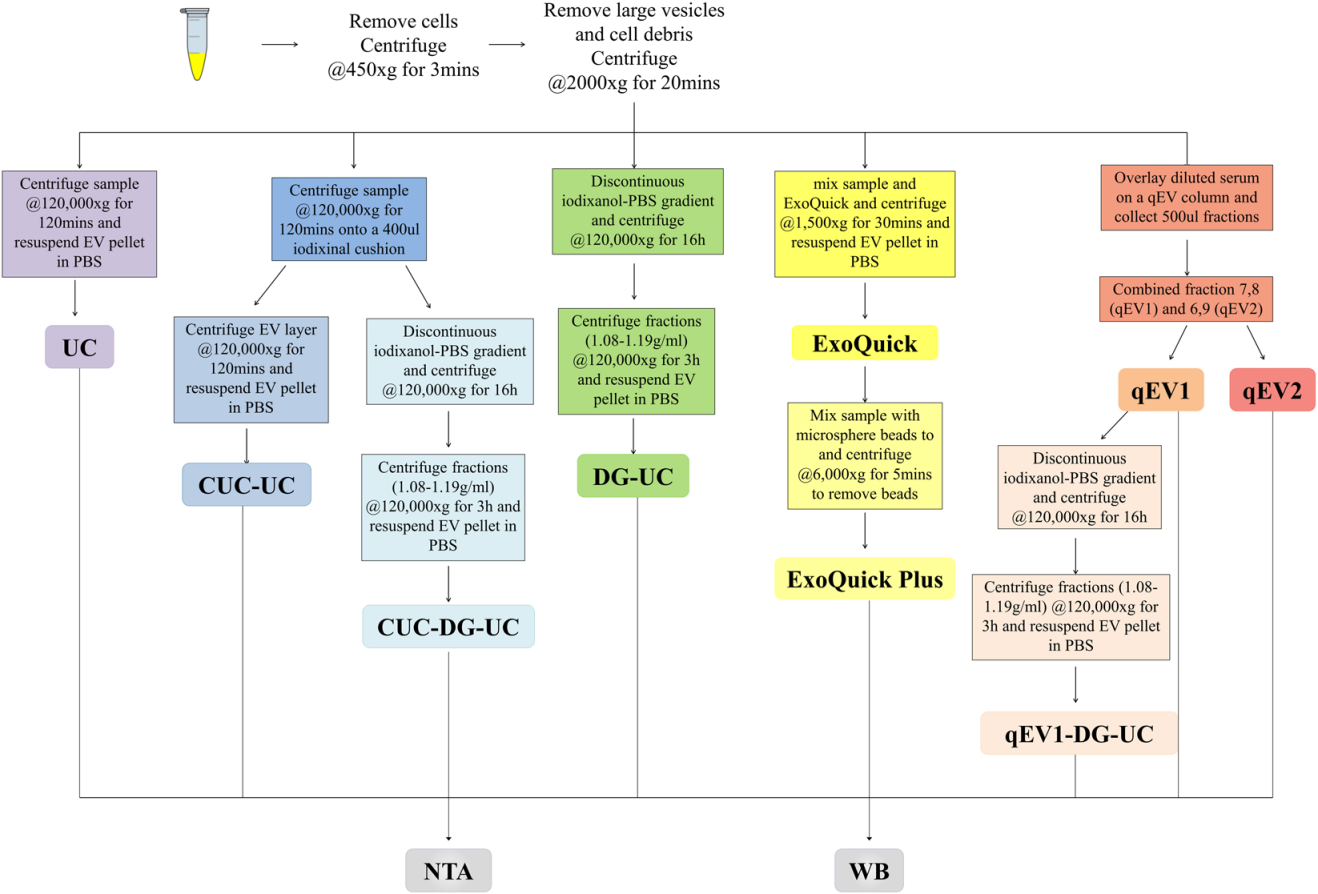
Schematic summary of EV isolation and downstream analyses. EVs were isolated from human serum using five different methods alone or in combination and characterized by Western blot (WB), nanoparticle tracking analysis (NTA).

In this study the EV isolation efficacy of these methods, used alone or in combination, from 200 µl of human serum (Fig. 1) was assessed via comprehensive nanoparticle tracking analysis (NTA)-based assessment of particle size and concentration. 200 µl of human serum was used in order to mimic the use of biobanked serum samples which were stored in 100–500 µl volumes. Furthermore, EVs isolated using the various approaches were examined to determine the best method for separation of sEVs from non-EV material in human serum. EV isolation was confirmed by transmission electron microscopy and the presence of EV protein markers, CD63 and TSG101. EV to protein ratio was used to measure non-EV protein contamination, whereas the presence of lipoprotein particles was assessed by Western blot analysis using the lipoprotein markers, APOB and APOE. This study outlines a variety of approaches that can be applied in EVs studies using human serum, each with advantages and disadvantages. Overall, the study reveals that the choice of EV isolation method(s) used greatly influences the depletion of lipoproteins and protein contaminants and the overall yield of EVs.

## Materials and Methods

### Serum isolation

Human serum was obtained with informed consent from five healthy non-fasting adult volunteers and all experiments were performed in accordance with relevant guidelines and regulations. The experimental protocols were approved by St. James's hospital Research Ethics Committee. Blood was drawn through a butterfly needle collection set (BD Vacutainer, Becton, Dickinson & Co., Franklin Lakes, NJ) and processed according to manufacturer’s instructions. In brief, samples were rested upright for 60 min to allow RBCs to clot. The RBC clot was subsequently pelleted by centrifugation at 1300 g for 10 min and serum was aspirated. The supernatant was centrifuged at 2000 g for 20 min in an SX4250 centrifuge to pellet cell fragments and other debris. Serum samples were pooled and stored in 200 µl aliquots at −80 °C prior to analysis.

### NTA analysis

Particle number and size distribution in serum samples was determined by nanoparticle tracking analysis (NTA) using a NanoSight NS300 system (Malvern Technologies, Malvern, UK) configured with a 488 nm laser and a high sensitivity scientific CMOS camera. Samples were diluted (serum 1:500) in particle-free PBS (Gibco, Waltham, MA, USA) to an acceptable concentration, according to the manufacturer’s recommendations. Samples were analysed under constant flow conditions (flow rate = 50) at 25 °C^43–45^ and 15 × 60 s videos were captured with a camera level of 14. Data was analysed using NTA 3.1.54 software with a detection threshold of 5 and using a bin size of 2.

### Transmission electron microscopy

Isolated EVs were deposited onto formvar/carbon-coated copper EM grids (10 μl on each grid) for 20 min. The vesicle-coated grids were washed three times with PBS and then fixed with 2.5% glutaraldehyde for 10 min. After washing in 3 drops of distilled water, the grids were stained with 2% uranyl acetate for 15 min and air dried for 20 min. Transmission electron microscopy was performed using a FEI Tecnai 120 microscope operating at an accelerating voltage of 120 keV.

### Exoquick plus

200 μl of the serum was diluted to 250 μl with particle-free PBS (Gibco, Waltham, MA, USA) before adding 63 μl of ExoQuick and incubated on ice for 30 min. The ExoQuick/serum mixture was centrifuged at 1500 g for 30 min. The supernatant was removed and the EV pellet was centrifuged at 1500 g for 5 min to remove residual ExoQuick solution. The EV pellet was resuspended in 250 μl of resuspension buffer and the protein concentration was determined by Bradford assay. 1 unit of the microsphere beads was washed with resuspension buffer three times, by vortexing the solution for 3 min, followed by centrifugation at 3000 g for 3 min. The beads were resuspended in 400 µl of resuspension buffer and added to the EVs based on their protein concentrations (2 µl beads solutions per 25 µg of protein). The samples were mixed on an inverting shaker for 15 min and centrifuged for 5 min at 6000 g to remove the beads.

### Size exclusion chromatography

IZON qEV original size exclusion columns were removed from 4 °C and the 20% ethanol storage solution was allowed to run through the column followed by 20 ml particle-free PBS. Serum samples were diluted to 500 µl with particle-free PBS and the sample was overlaid on the qEV size exclusion column followed by elution with particle-free PBS. The flowthrough was collected in 500 µl fractions, and fractions 7 and 8 were combined into qEV1 and fractions 6 and 9 were combined into qEV2 for analysis. The column was washed by running through 50 ml particle-free PBS followed by 20% ethanol storage solution.

### Cushion ultracentrifugation (CUC) and ultracentrifugation (UC)

All ultracentrifugations were performed at 120000 g AVG in Beckman Coulter Optima L-100 XP ultracentrifuge (stopping without break), with centrifugation durations based on a “50 nm cut-off size” adjustment to the centrifugation duration for each rotor as described in Livshits *et al*. 2015, with additional 5 min added to allow the rotor to come up to speed^46^. Serum samples were defrosted and transferred to a 5 ml Beckman ultracentrifuge tube (Prod. No. 344057). The serum was then diluted to 4.6 ml with particle-free PBS and 400 µl of 40% iodixanol-PBS was pipetted into the bottom of the tube for Cushion ultracentrifugation (CUC) while the serum was diluted to 5 ml with particle-free PBS for ultracentrifugation (UC). The tubes were centrifuged at 120000 g (RCF avg, 35600 rpm) for 2 hours at 4 °C, using Beckman Coulter SW55ti rotor. For UC, the supernatant was removed and the EV pellet was resuspended in 50–100 µl residual PBS. For CUC the EV sample was resuspended in 5 ml particle-free PBS and centrifuged at 120000 g (RCF avg, 35600 rpm) for 2 hours at 4 °C, using Beckman Coulter SW55ti rotor. The supernatant was removed and the EV pellet was resuspended in 50–100 µl residual PBS.

### Iodixanol density gradient centrifugation

Density gradient centrifugation was preformed using a modified protocol from Paolini *et al*.^47^. A 54% iodixanol-PBS working solution was prepared by diluting a stock solution of OptiPrep™ (60% (w/v) aqueous iodixanol from Axis-Shield PoC, Norway) with 10x particle-free PBS (Gibco, Waltham, MA, USA). Iodixanol solutions (41% (w/v), 35% (w/v), 23% (w/v) and 14% (w/v)) were prepared by diluting the 54% iodixanol-PBS working solution in 1x particle-free PBS (Gibco, Waltham, MA, USA). To form the gradient, firstly a homogenous 41% (w/v) base layer of the gradient (estimated density ~1.224 g/ml) was produced by adding 2.278 ml of the 54% iodixanol-PBS working solution to a 14 × 89 mm thinwall, ultra-Clear™ tube (Beckman Coulter), together with either serum or EVs isolated by cushion ultracentrifugation (CUC) or size exclusion chromatography (qEV1) diluted to 722 µl with particle-free PBS. Next, 3 ml 35% (w/v) iodixanol (estimated density ~1.192 g/ml), 3 ml 23% (w/v) iodixanol (estimated density ~1.128 g/ml), and 2 ml 14% iodixanol (estimated density ~1.08 g/ml) were layered successively on top of the vesicle suspension. Centrifugation was performed at 120000 g (RCF avg) for 16 h at 4 °C in Beckman Coulter Optima L-100 XP ultracentrifuge, using Beckman Coulter SW41ti rotor (28,500 rpm, stopping without break). Fractions (~300 µl) were collected from the top of the tube using a peristaltic pump. 50 µl of each fraction was diluted 1 in 4 with particle-free PBS and absorbance was measured at 340 against an iodixanol standard curve to determine the fraction density. The fractions with densities between 1.08–1.19 g/ml were combined and diluted to a density <1.03 g/ml with particle-free PBS and the diluted fractions were centrifuged at 120000 g (RCF avg, 35600 rpm using Beckman Coulter SW41ti rotor, or 31300 rpm using Beckman Coulter SW32ti rotor) for 3 hours at 4 °C in Beckman Coulter Optima L-100 XP ultracentrifuge (stopping without break). The supernatant was removed and the EV pellets were resuspended in 50–100 µl residual PBS.

### Flow cytometry analysis

Flow cytometry analysis was performed on the Beckman Coulter CytoFLEX LX Flow Cytometer. The flow cytometer was equipped with 375 nm, 405 nm, 488 nm, 561 nm, 638 nm, and 808 nm lasers to detect up to 21 fluorescence parameters. For daily calibration of the flow cytometer fluorescent polystyrene beads (Megamix FSC & SSC Plus, BioCytex, Marseille, FRA) were used in sizes of 100, 160, 200, 240, 300, 500, and 900 nm. The VSSC and SSC threshold was set as the trigger channel below the 0.1 µm bead population which gave us an acceptable noise of about 30–200 events/sec. A rectangular gate was set on the VSSC-H log x RSSC-H log cytogram containing the 100 nm and 240 nm bead populations and defined as ‘100 nm–240 nm Megamix gate’ followed by a “stable time gate” set on the time histogram in order to identify the microparticle region (Fig. S1A–1B). To avoid swarm effects each was serially diluted from 1:2 to 1:500 and measured with a flow rate of 10 µL/min prior to antibody labelling. EVs were labelled with 0.05ul anti-CD63-PE (H5C6, BD Bioscience) or anti-CD147-APC (MEM-M6/1, Thermo Scientific) in 100ul PBS for 30 mins on ice in the dark. To avoid false positive events, all antibodies used were run in PBS alone to ensure antibody clumps were not present. To avoid carry‐over effects between each sample measurement we performed a washing step with filtered double distilled water for 1 min at an increased flow rate of 60 µL/min. EV lysis was performed by incubating PBS-diluted EVs in 0.05% Triton™ X-100 for 30 min at room temperature.

### EV protein isolation and quantification

EVs were lysed in an appropriate volume of 5x RIPA buffer [50 mM Tris-Cl pH 7.4, 750 mM NaCl, 0.5% Sodium Deoxycholate, 0.5% SDS, 5% Triton, 1 mM Sodium Orthovanadate, 50 mM NaF, 5 µg/ml Pepstatin, 5 µg/ml Aprotinin, 5 µg/ml Leupeptin] on ice for 30 min followed by centrifugation at 14000 g for 10 min. 1 volume of 100% (w/v) TCA was added to 4 volumes of the sample and incubated for 10 min at 4 °C. After centrifugation at 14000 g for 5 min the supernatant was removed and the pellet was washed twice with cold acetone. The pellets were heated to 95 °C for 5–10 min and the dried pellets were resuspended in 50 µl of 6 M urea in 50 mM ammonium bicarbonate. The protein content was assessed using the micro-BCA assay (Walker, 1994). 5 μL of sample was added to a 96-well microplate followed by 100 μL of BCA reagent (Pierce, USA). The plate was incubated in the dark for 30 min at 37 °C and absorbance was measured at 560 nm and protein concentration was determined from a BSA standard curve.

### SDS-Polyacrylamide Gel Electrophoresis (SDS-PAGE) and Western Blot Analysis

Protein (4 µg) was mixed with 4X Laemmli buffer (750 mM Tris-HCl pH6.8, 5% SDS, 40% glycerol and 80 mM DTT) and heated to 95 °C for 5 min. Protein was resolved on fixed-percentage (12%) polyacrylamide resolving gels overlain by stacking gels (1.5 mm thick). Gels were run in Tris-Glycine running buffer at 80 mA for 70 min. Resolved proteins were then transferred to 0.45 µm nitrocellulose membrane using a mini-Protean II blotting system at 110 V constant voltage for 70 min. The membranes were blocked for 1 h at RT in 1X TBS containing 5% (w/v) bovine serum albumin. Proteins were detected by incubation with primary antibodies (CD63 (H5C6-BD bioscience, 556019, 1/500) CD63 (H193-Santa Cruz Biotechnology, sc-15363, 1/500) TSG101 (ABCAM, ab125011, 1/500) APOE (Santa Cruz Biotechnology, sc-390925, 1/1000) APOB (Santa Cruz Biotechnology, sc13538, 1/1000)) in blocking solution overnight at 4 °C. Following three 5 min washes of TBS with 0.1% Tween-20 (TBS-T), membranes were incubated in the appropriate dilution of IRDye800-conjugated goat anti-rabbit IgG and IRDye680-conjugated goat anti-mouse IgG secondary antibodies (LI-COR Biosciences) diluted in 5% blocking solution for 1 h at RT. The blots were then washed six times, alternating between TBS and TBST. Proteins were visualized by scanning the membrane on an Odyssey Infrared Imaging System (LI-COR Biosciences) with both 700- and 800-nm channels.

## Results

### Particle size distribution of human serum EVs differ based on the method of isolation

**Figure 2:**
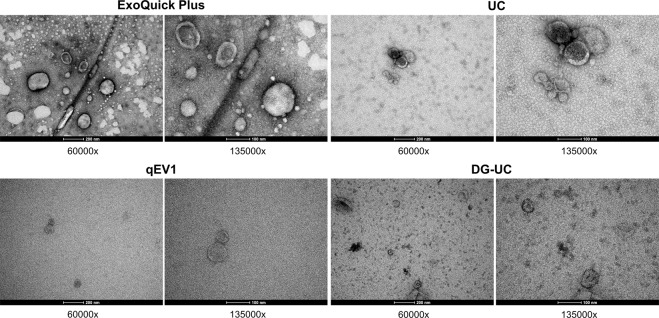
Transmission electron microscopic (TEM) visualization of the EVs isolated from pooled human serum. Representative electron microscopy images of EVs isolated by ExoQuick Plus, ultracentrifugation (UC), size exclusion chromatography (qEV1), or density gradient ultracentrifugation (DG-UC), from pooled human serum. 60k and 135k magnifications are shown.

**Figure 3:**
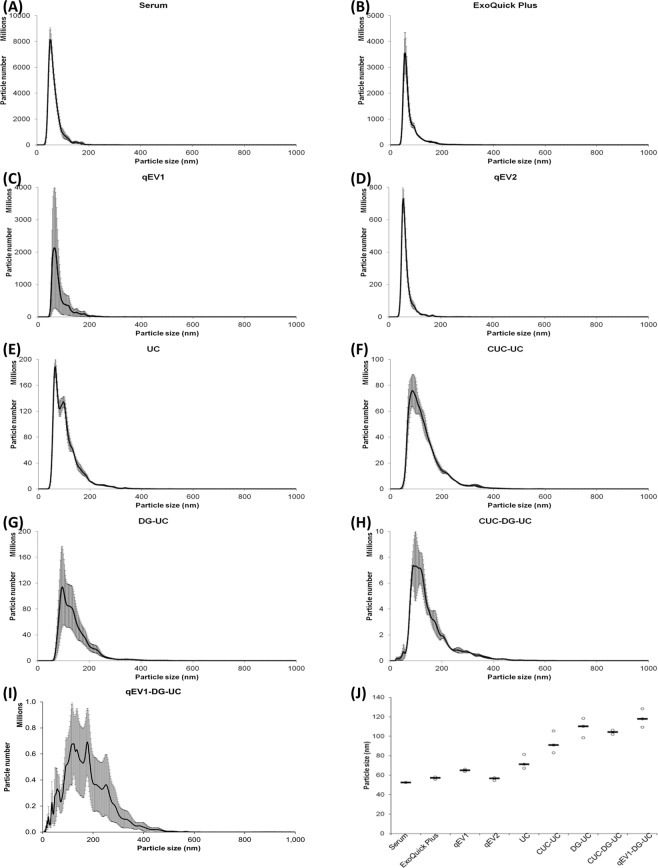
Nanosight analysis of particle size distribution of the EVs isolated from pooled human serum. Nanoparticle tracking analysis (NTA) of the total vesicles isolated from 200 µl of serum by each method alone or in combination. (**A**–**I**) Line graphs corresponding to average number and size of particles isolated by each method, calculated from the mean of 15 videos per isolation.) (**J**) Dot plots representing the variation in the modal size of the vesicle isolated by each method. Black lines represent medians. N = 3 isolations.

Human serum contains soluble protein and lipoprotein particles that overlap with EVs in size and density (Table 1). An aim of this study was to assess the suitability of different EV-enrichment methods for the separation of EVs from non-EV protein and lipoprotein particles from a small volume of human serum. EVs were isolated from 200 µl of pooled human serum using differential centrifugation followed by either; ultracentrifugation (UC), polymer-based precipitation using Exoquick plus, size exclusion chromatography (SEC) using qEV columns or iodixanol density gradient centrifugation (DG) followed by ultracentrifugation. Using electron microscopy, we were able to visualize cup-shaped vesicles with morphology and size compatible with EVs, in addition to particles of smaller sizes (Fig. 2). The size distribution of particles present after each EV isolation method, either alone in or in combination, was determined by NTA (Fig. 3B–I), with NTA of the pooled serum was used as a reference (Fig. 3A) and used to determine the extent of lipoproteins present (Table 2).

**Table 2:** Nanoparticle tracking analysis and protein concentration of EVs isolated from human serum using the different methods. Data included in this table represent the mean readings of three experimental replicates that were extracted, diluted, and measured separately.

Isolation Technique	Modal size (nm)	Median size (nm)	Particle number	Total Protein (µg)
unprocessed serum	52.4 ± 0.7	58.8 ± 0.4	1.44 × 10^11^ ± 2.43 × 10^10^	—
ExoQuick	—		—	2205.7 ± 12.0
ExoQuick plus	57.0 ± 0.7	64.2 ± 3.0	5.59 × 10^10^ ± 1.01 × 10^10^	911.0 ± 98.2
qEV1	64.9 ± 0.7	73.4 ± 2.3	4.20 × 10^10^ ± 3.63 × 10^10^	17.1 ± 4.3
qEV2	56.2 ± 0.9	58.6 ± 1.1	9.46 × 10^9^ ± 1.33 × 10^9^	19.4 ± 8.6
UC	73.2 ± 4.2	92.9 ± 2.4	6.27 × 10^9^ ± 4.66 × 10^8^	772.3 ± 19.8
CUC-UC	93.2 ± 6.6	115.2 ± 3.7	3.80 × 10^9^ ± 5.21 × 10^8^	543.5 ± 19.9
DG-UC	109.0 ± 5.8	130.7 ± 5.9	4.93 × 10^9^ ± 2.07 × 10^9^	31.1 ± 14.9
CUC-DG-UC	104.2 ± 1.3	127.6 ± 3.2	4.02 × 10^8^ ± 6.00 × 10^7^	22.1 ± 2.1
qEV1-DG-UC	118.5 ± 5.5	152.9 ± 6.6	6.03 × 10^7^ ± 3.06 × 10^7^	17.1 ± 0.9

Data intervals represent the SEM. UC, ultracentrifugation; ExoQuick: Exoquick Serum Exosome Precipitation Solution (System Biosciences, Mountain view, CA).

The modal size of the particles isolated from serum by each method are shown in Fig. 3J. EVs isolated using ExoQuick Plus, and qEV2 yielded modal particle sizes in the range 52.4–57 nm, which is similar to serum, while EVs isolated by qEV1 and ultracentrifugation had an increased modal particle sizes of 64.9 nm (qEV1), 73.2 nm (UC) and 93.2 nm (CUC-UC). The highest modal particle sizes, which are in the range 104.2–118.5 nm, were observed in EVs isolated by methods using density gradient centrifugation (Fig. 3J, Table 2). This increased modal size is also accompanied by a broad size distribution as a variety of particle sizes between 60–150 nm become more abundant relative to <60 nm particles. These <60 nm particles detected by the Nanosight could be due to protein aggregates as an abundance of <60 nm particles are observed in later size exclusion fractions as protein concentration increases (Supplementary Table 1) or could indicate the presence of LDL and VLDL^48^.

### The yield of sEVs (61–150 nm) isolated from human serum differs depending of method of isolation

**Figure 4:**
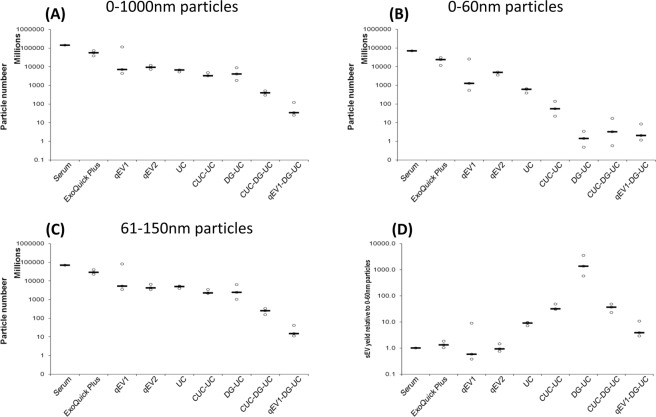
Nanosight analysis of particle number of the EVs isolated from pooled human serum. Nanoparticle tracking analysis (NTA) of the total vesicles isolated from 200 µl of serum by each method alone or in combination. Dot plots corresponding to average numbers of vesicles between (**A**) 0–1000 nm, (**B**) 0–60 nm, and (**C**) 61–150 nm isolated by each method, calculated from the mean of 15 videos per isolation. (**D**) Dot plots corresponding to sEV (61–150 nm vesicle) number/ 0–60 nm vesicle number isolated by each method. Black lines represent medians. N = 3 isolations.

The total number of particles isolated from 200 µl human serum using the different EV isolation methods was measured by NTA. 200 μl of human serum was found to contain 1.4 × 10^11^ particles, the application of Exoquick plus and qEV1 for EV enrichment yielded the greatest number of total particles, with 5.6 × 10^10^ and 4.2 × 10^10^ particles isolated respectively (Fig. 4A). The application of qEV2, UC, CUC-UC and DG-UC yielded between 3.8 × 10^9^ and 9.5 × 10^9^ particles. The use of additional techniques before DG-UC resulted in a significant decrease in particle yield. Specifically, CUC-DG-UC and qEV1-DG-UC yielded 4 × 10^8^ particles and 6 × 10^7^ particles respectively (Fig. 4A). The decrease in particle number observed in this study (Fig. 4A) together with the increase in modal size (Fig. 3J) is likely due to a reduction in either protein aggregates (Supplementary Table 1) or number of smaller particles such as lipoproteins. Mørk *et al*., reported a similar trend following immunodepletion of LDL and VLDL from platelet-free plasma resulting in a 62% decrease in particle number and a 37 nm increase in mean particle diameter by NTA^48^.

The effect that each EV isolation method had on small particles (0–150 nm) was investigated. NTA analysis revealed a large proportion of small particles in the serum indicated by a median particle size of 58.8 nm in serum (Table 2). Due to the sensitivity limits of the NS3000, 46–70 nm is reported to be the minimum detectable particle size for NTA (depending on the refractive index of the particle), as a result the actual size and concentration of particles <60 nm may be underestimated by the NTA^49^. However, considering that the minimum accurate bin size for NTA is 2 nm, particles were grouped into two populations, 0–60 nm which is equal to or less than the median, and 61–150 nm which is greater than the median.

Comparison of the number of small particles <60 nm detected with each method revealed that Exoquick had the highest yield with 2.4 × 10^10^ < 60 nm particles detected compared to the 7 × 10^10^ < 60 nm particles measured in serum. Size exclusion chromatography fractions qEV1 and qEV2 yielded 1.3 × 10^9^ and 4.9 × 10^9^ < 60 nm particles respectively. However ultracentrifugation-based methods (UC and CUC), and density gradient centrifugation-based methods (DG-UC, CUC-DG-UC and qEV1-DG-UC) had significantly lower levels of <60 nm particles, with UC and CUC yielding 6.2 × 10^8^ and 5.5 × 10^7^ < 60 nm particles respectively, density gradient centrifugation-based methods yielding between 1.8 × 10^6^ and 6.8 × 10^6^ < 60 nm particles methods (Fig. 4B).

Figure 4C shows the number of particles isolated of between 61–150 nm using each method. The data obtained follows a similar trend as observed for 0–1000 nm particles (Fig. 4A). The yield of 61–150 nm particles (sEVs) relative to <60 nm particles (sEVs/<60 nm) was used to determine the best method for isolation of the sEV population (60–150 nm) with the exclusion of particles <60 nm, which based on their size, is likely to be a combination of protein aggregates and lipoproteins (Fig. 4D). While DG-UC had the greatest relative yield of sEVs based on the sEVs/<60 nm ratio (Fig. 4D), the use of additional techniques before DG-UC reduced the sEVs/<60 nm ratio due to a reduced yield of 61–150 nm particles (Fig. 4C). The relative yield of sEVs/<60 nm particles was also higher in CUC-UC relative to UC alone, indicating that repeated ultracentrifugation steps could also reduce the number of <60 nm particles co-isolated with the 61–150 nm sEVs. However, the reduction in the sEV yield from 5 × 10^9^ particles in UC to 2.3 × 10^9^ particles in CUC-UC (Fig. 4C) implies there is a limit to the number of ultracentrifugation steps that can performed due to loss in EV yield.

### Quantification of protein levels present after isolation of EVs from human serum using different isolation methods

**Figure 5:**
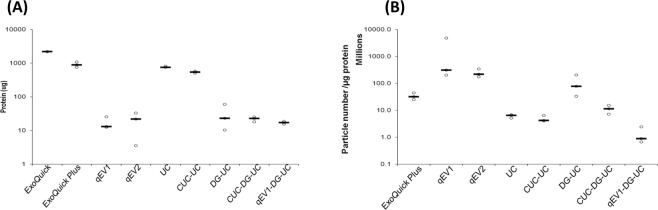
Serum protein co-isolated with EVs isolated from pooled human serum. (**A**) Dot plots representing the total amount of protein present in the EV isolations by each method alone or in combination was determined by BCA assay. N = 3 isolations (**B**) Dot plots corresponding to 61–150 nm vesicle number/µg of protein isolated by each method. Vesicle number was determined by nanoparticle tracking analysis (NTA) of 15 videos per isolation. Black lines represent medians. N = 3 isolations.

EVs isolated from 200 µl serum using the different methods were lysed and the total protein content (EV and soluble protein) present in each fraction was determined by BCA assay (Fig. 5A). The amount of protein obtained differed significantly across the methods used and ranged from 17.1 ± 4.3 μg using qEV1 to 911 ± 98.2 μg using ExoQuick plus. ExoQuick plus and ultracentrifugation (alone or following CUC) captured a 40–50-fold increase in protein compared to SEC and density gradient ultracentrifugation-based protocols (Fig. 5A). The amount of protein contamination present in each isolation was determined by calculating the ratio of particle count and protein concentration as described by Webber and Clayton, 2013 in order to assess the level of contaminating non-EV protein retrieved using each method, however large numbers of protein aggregates may contribute to the particle counts (Supplementary Table 1) potentially skewing the ratio^50^. Results revealed that qEV1, qEV2 and DG-UC yielded higher particle to protein ratios than all other methods, indicating less soluble protein co-isolated (Fig. 5B). In contrast, the use of CUC or qEV1 before DG-UC resulted in a lower particle to protein ratio, which can be explained by the lower particle yield obtained with these methods (Fig. 4A), while the low particle to protein ratio observed following ultracentrifugation was due to a high level of co-isolated protein (Fig. 5B). The use of CUC-UC revealed that the additional ultracentrifugation step reduced the level of co-isolated protein, however, the particle to protein ratio was unchanged (Fig. 5B) due to a similar decrease in particle yield (Fig. 4A).

### Comparison of EV enrichment using different isolation methods

**Figure 6:**
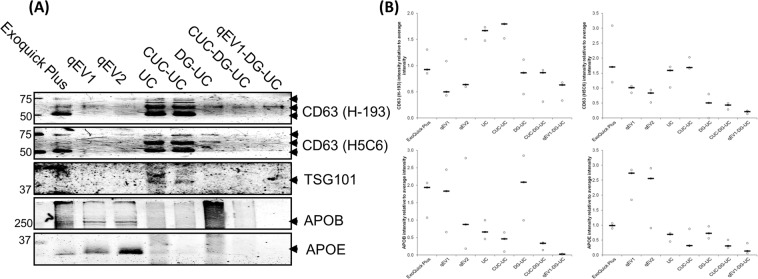
Western blot analysis of EV markers, CD63 and TSG101, and lipoprotein markers, APOB, and APOE. (**A**) 4 µg of vesicular protein isolated from 200 µl human serum using each method alone or in combination were separated by SDS–PAGE and immunoblotted with anti-human CD63 (H-193 and H5C6), TSG101, APOE and APOB antibodies. Representative images are shown. (**B**) Graph: Densitometric quantitation of the distribution of CD63, TSG101, APOE and APOB protein expression in each method. Black lines represent medians. N = 3 isolations. Arbitrary Units (AU) = (signal intensity method)/Sum (signal intensity method1–8).

Comparison of EV enrichment was performed by analysis of the EV protein markers, CD63 and TSG101 by Western blot (Fig. 6A). CD63 was detected using two antibodies (clone H-193 and H5C6) as CD63 is reported at multiple sizes^51^ potentially due to differential glycosylation. Three protein bands 50 kDa, 60 kDa and 75 kDa were detected by both CD63 antibodies at different intensities across the different isolation methods. EVs isolated by ExoQuick plus and ultracentrifugation showed high signal intensities for CD63, whereas EV samples isolated using SEC-based and density gradient centrifugation had less CD63 protein present, however, a greater proportion of the CD63 protein is represented as a 60 kDa glycosylated protein whereas the EVs isolated by ExoQuick plus and ultracentrifugation had 50, 60, and 75 kDa forms of CD63 protein. A fourth band was detected in EVs isolated by ultracentrifugation using the H-193 but not by H5C6 antibody clone, which may represent a distinct vesicle population that was not isolated by the other methods. However, we cannot exclude the possibility that it represents non-specific binding of the H-193 antibody to a contaminating protein isolated by this method. TSG101 was also detected in EV samples isolated by ultracentrifugation, however, it was only poorly detected in EVs isolated by the other methods (Fig. 6). The presence of EV markers was also confirmed by flow cytometry analysis of EVs labelled with anti-CD63-PE (H5C6, BD Bioscience) or CD147-APC (EMMPRIN, MEM-M6/1, Thermo Scientific) (Supplementary Fig. 1). While all isolation methods showed increased median staining intensity with increased rSD for CD63 (Supplementary Table. 2) and CD147 (Supplementary Table. 3) relative to unstained and EV lysed controls, ExoQuick plus, UC, and CUC-UC showed the greatest increase in median staining intensity relative to the other methods.

### Comparison of lipoprotein burden using different isolation methods

The relative lipoprotein burden was determined by analysis of the lipoprotein markers APOB (chylomicrons, VLDL, IDL, and LDL) and APOE (chylomicrons, VLDL and HDL) by Western blot analysis of equal amount of protein isolated by each method (Fig. 6). APOB and APOE were most abundant in EVs isolated using ExoQuick plus and SEC, with increased APOE detected in qEV2 relative to qEV1 as more HDL lipoprotein particles are eluted from the column. APOB and APOE levels were lower in CUC-UC relative to UC and in CUC-DG-UC and qEV1-DG-UC relative to DG-UC suggesting the combination of 2 or 3 isolation methods resulted in concomitant reduction in quantity of lipoproteins co-isolated. The levels of APOB and APOE appear to be disproportionally larger in qEV1 and DG-UC compared to either UC or CUC-UC by Western blot analysis relative to the NTA results for <60 nm particles (Fig. 4B), which is in part due to the use of equal protein loading for Western blot analysis and the greater particle/protein ratio of the qEV1 and DG-UC EV isolations (Fig. 5B). Chylomicrons or lipoprotein-EV aggregates^52^ could contribute to APOB and APOE protein levels in the isolations without affecting particle sizes <60 nm.

## Discussion

EVs are released into the bloodstream by cells under normal and pathological conditions and carry RNA, lipids, and proteins from their host cells that can represent the molecular composition of the parental cell. For example, Shao *et al*. found that exosomal mRNA levels of the drug resistance markers MGMT (O(6)-methylguanine DNA methyltransferase) and APNG (alkylpurine-DNA-N-glycosylase) correlate with mRNA levels found in parental glioblastoma multiforme cell lines^53^. Tumour derived exosomes can also contribute to the establishment of a pre-metastatic niche as a result of the exosomes being taken up by endothelial cells, inducing the expression of VEGF, VEGF-R1 and ICAM-1 resulting in enhanced angiogenesis and increased adhesion between the cancer and endothelial cells^54^.

These functions of EVs in modifying distant microenvironments to promote metastasis as well as several studies reporting differentially packaged RNA and protein in cancer cell line-derived EVs highlights their potential as cancer biomarkers with diagnostic and prognostic value^53,55,56^. For example, the EV associated miRNAs miR-222-3p is detected in human serum and is associated with poor prognosis in NSCLC patients^57^. Despite this, the potential use of other RNA and protein targets identified in EVs secreted by cancer cell lines i*n vitro* is hindered by challenges in biomarker validation in patient samples. Although affinity capture of cancer specific EVs from patient serum has shown some success in ovarian cancer^58^, there is currently no standardised method for isolating and purifying circulating EVs from human serum.

Commonly used EV isolation methods include ultracentrifugation, density gradient centrifugation, size exclusion chromatography, and polymer-based precipitation, with each varying in yield of EVs, the depletion of lipoproteins and protein contaminants, labour-intensity, and cost of the procedure. Other challenges include a high abundance of serum proteins, such as albumin and globulins, and non-EV lipid particles such as chylomicrons and lipoprotein particles that can interfere with particle counts, biomarker analysis with some lipoproteins such as HDL reported containing miRNAs^30–32^. Furthermore the levels of chylomicrons and lipoprotein particles can vary greatly from individual to individual and are influenced by diet, genetics and race^33–38^ adding further complexity to biomarker validation studies. Therefore, separation of serum EVs from soluble proteins and non-EV lipid particles is critically important for the development of strategies for biomarker discovery and validation.

In this study, we performed a quantitative and qualitative comparison of EV populations isolated from 200 μl of human serum using a number of commonly used methods to determine the best approach for isolation of high EV yields from low sample volumes. Moreover, we measured the presence of contaminating soluble protein and lipoprotein particles in the EV samples at the end of each isolation procedure alone, or following a combination of isolation methods. In our screening, we included ultracentrifugation (UC), polymer-based precipitation using Exoquick plus, size exclusion chromatography (SEC) using qEV columns and ultracentrifugation combined with iodixanol density gradient centrifugation. Particle size and number was determined using NTA. Unsurprisingly, all methods used in this study successfully isolated particles in the size range of small EVs between 61 nm and 150 nm. There was a significant decrease in <60 nm particles detected as well as an increase in the modal particle size from 52.4 nm to between 73.2 nm and 118.5 nm following either ultracentrifugation or density gradation ultracentrifugation (Figs. 3J, 4B). Particles <60 nm represent vesicles with a bin size less than or equal to the median particle size detected by NTA in serum (58.8 nm) and correspond to the reported sizes of LDL, VLDL, as well as protein aggregates. While EVs can be smaller than 60 nm, it has been reported that lipoprotein particles are several fold more abundant than EVs in human serum^52,59,60^. Furthermore since several lipoproteins particles and protein aggregates fall below the minimum detectable particle size for NTA (46–70 nm depending on the refractive index of the particle), the actual number of particles <60 nm may be underestimated by the NTA^49^. A recent study by Mørk *et al*. has shown a similar change in mean particle diameter from 54.7 nm to 91.7 nm after immunodepletion of LDL and VLDL from platelet-free plasma^48^ supporting the notion that LDL and VLDL lipoproteins contribute to the population of <60 nm particles detected by NTA.

NTA revealed the sEV yield was greatest for ExoQuick plus and size exclusion chromatography (qEV1), however the high yield of sEVs by qEV1 was not reflected in CD63 protein levels detected by western blot. Density gradient centrifugation (DG) yielded the greatest number of sEVs (61–150 nm particles) relative to 0–60 nm particles, which was followed by iodixanol cushion ultracentrifugation and ultracentrifugation (CUC-UC). In contrast there was a greater amount of CD63 protein detected in the EV sample isolated by CUC-UC relative to that isolated by DG. The greater amount of CD63 detected in samples with greater amounts of non-EV protein such as ExoQuick plus, UC and CUC-UC may be due to additional CD63 protein attached to vesicular membrane fragments that are not quantified by NTA or a cleaved extracellular domain of CD63 present in solution. Alternatively, it may be due to differential enrichment of CD63 positive and negative vesicles. In support of this, it has been demonstrated that EV isolates are a heterogeneous mixture of EV subpopulations with specific protein profiles^61,62^, suggesting that isolation methods could be biased towards only partially overlapping EV populations.

In addition to differences in the amount of CD63 detected, the western blot data revealed that the proportions of each of the 3 glycosylated forms of CD63 detected in the EV samples varied depending on the isolation method, with the 50 kDa protein predominantly found in EV samples isolated using the Exoquick plus, UC and CUC-UC. A 60 kDa form of CD63 and a weaker 75 kDa form were detected in all EV samples, while a fourth CD63 protein of approximately 70 kDa was detected only in EVs isolated by UC and CUC-UC. TSG101 was only weakly detected in EVs isolated using UC and CUC-UC, and was not detected in EVs isolated using the other methods, which is consistent with recent studies reporting the absence of TSG101 in plasma^63^ and serum EVs^64^.

EV samples obtained using qEV1 and qEV2 had the highest levels of the VLDL and HDL marker APOE, while ExoQuick plus, qEV1 and DG-UC had the highest levels of the VLDL, IDL and LDL marker APOB. Although APOB positive particles were detected by DG-UC, these were removed partially by the addition of a cushion ultracentrifugation before density gradient centrifugation. The higher amounts of APOE detected in qEV1 and qEV2 is probably due to the qEV columns being more efficient at removing protein than VLDL and HDL from the sample with this lower protein concentration resulting in a greater proportion of the sample being analysed. This may account for the difference between the <60 nm particle count by NTA and the APOB and APOE western blot analysis. Alternatively, the formation of chylomicrons or lipoprotein-EV aggregates^52^ could be contributing to APOB and APOE protein levels in the isolations without affecting the <60 nm NTA. It was found that the combination of size exclusion chromatography, density gradient centrifugation and ultracentrifugation (qEV1-DG-UC) provided EV samples with the greatest depletion of lipoproteins, containing the lowest levels of APOB and APOE positive particles; however, the total particle yield is low, therefore this isolation procedure may not be compatible with all downstream applications.

In this study we have highlighted some of the challenges and limitations in isolating EVs from small volumes of human serum that should be considered for retrospective studies where small volume biobanked serum is the source of EVs. Firstly, the abundance of the EV marker, CD63, detected by western blot was low but tended to correlate with the amount of protein in the sample and not the NTA particle counts. Flow cytometry detection of CD63 is an alternative approach to confirm EVs. Furthermore, is it likely that NTA overestimates the amount of EVs due to the presence of lipoproteins and protein aggregates and highlights a limitation in using particles/ug to determine EVs/ug when dealing with human serum.

In conclusion, we have conducted a study to compare the EV yield obtained from a small volume of human serum, as well as the depletion of lipoproteins and protein contaminants, using a number of commonly used methods. The ability to separate EVs from non-EV protein and different groups of lipoproteins varied considerably across the methods tested. The sequential use of two or more isolation methods greatly improved depletion of lipoprotein and protein contaminants; however, this was accompanied by a significant decrease of overall EV number. Therefore, the choice of EV isolation method used should depend on the amount of starting material together with the downstream application, and will be influenced by the need to remove all or only distinct groups of non-EV serum components.

## Supplementary information

Supplementary material.
