# Supplementary material for: A comparison of methods for the isolation and separation of extracellular vesicles from protein and lipid particles in human serum
Source: Sci Rep. 2020 Jan 23;10:1039. doi: 10.1038/s41598-020-57497-7 (PMC6978318; doi:10.1038/s41598-020-57497-7)
Supplement: Supplementary file 1 — Supplementary material. [file 41598_2020_57497_MOESM1_ESM.pdf]

A comparison of methods for the isolation and separation of extracellular vesicles from protein and lipid particles in human serum

[1*]Brennan K, [2]Martin K, [3]FitzGerald SP, [4]O'Sullivan J, [1]Wu Y, [5]Blanco A, [2]Richardson C, and [1]Mc Gee MM

1. UCD School of Biomolecular & Biomedical Science, Conway Institute, University College Dublin (UCD), Belfield, Dublin 4, Ireland. 2. Randox Teoranta, Meenmore, Dungloe, Donegal, Ireland. 3. Randox Laboratories Ltd., Crumlin, Antrim, United Kingdom.4. Trinity Translational Medicine Institute (TTMI), Department of Surgery, Trinity College Dublin, St James's Hospital, Dublin, Ireland. 5. UCD Conway Flow Cytometry Core, Conway Institute, University College Dublin (UCD), Dublin, Ireland.

***Correspondence to: Kieran Brennan,** UCD School of Biomolecular & Biomedical Science**,** Conway Institute**,** University College Dublin**,** Belfield**,** Dublin 4**,** Ireland**. E-mail: k.brennan@ucd.ie**

Supplementary material

Size exclusion fraction number	Modal size (nm)	Median size (nm)	Particle number	Total Protein (µg)
Fraction 7,8 (qEV1)	78.2 +/- 3.4 nm	91.9 +/- 2.0 nm	7.86e+009 +/- 2.42e+008	14.07
Fraction 6,9 (qEV2)	68.7 +/- 3.4 nm	76.8 +/- 2.1 nm	5.39e+009 +/- 7.03e+008	31.65
Fraction 10	67.8 +/- 3.4 nm	71.5 +/- 0.9 nm	3.28e+009 +/- 1.30e+009	76.49
Fraction 11	60.0 +/- 1.9 nm	60.6 +/- 0.7 nm	7.4e+009 +/- 1.28e+009	169.99
Fraction 12	56.1 +/- 2.0 nm	59.3 +/- 0.6 nm	4.755e+009 +/- 5.45e+008	354.63
Fraction 13	55.2 +/- 1.8 nm	56.3 +/- 0.8 nm	4.875e+009 +/- 7.71e+008	699.88
Fraction 14	55.2 +/- 2.1 nm	56.9 +/- 0.6 nm	9.1e+009 +/- 2.26e+009	1181.12

Supplementary Table 1. Nanoparticle tracking analysis and protein concentration of size exclusion fractions. Data intervals represent the SEM.

Sample Name	Events/µL	Total events	Median Y585-PE-H	rSD Y585-PE-H
ExoQuick Plus unstained	3562.61	71252	313.3	94.9
ExoQuick Plus CD63-PE	1786.55	35731	357.8	150.6
ExoQuick Plus CD63-PE triton	3428.36	68567	321.6	114.2
UC unstained	4764.55	95290	308	95
UC CD63-PE	2458.15	49162	378.8	166.9
UC CD63-PE triton	2496.45	49929	319.7	116.8
CUC-UC unstained	5271.38	105427	312.5	94.6
CUC-UC CD63-PE	1266.1	25322	385	166.5
CUC-UC CD63-PE triton	1819.92	36398	321.8	114.3
qEV1 unstained	4245.25	84904	308.9	95.2
qEV1 CD63-PE	1743.71	34874	337.7	127.8
qEV1 CD63-PE triton	2467.58	49351	313.3	111
qEV2 unstained	3064.27	61285	310.4	93.1
qEV2 CD63-PE	2122.61	42452	338.1	133.4
qEV2 CD63-PE triton	3776.16	75523	318.7	113.9
DG-UC unstained	1866.1	37322	311	98
DG-UC CD63-PE	1430.98	28619	334.3	128.4
DG-UC CD63-PE triton	1889.6	37792	318.7	108.5
CUC-DG-UC unstained	2240.01	44800	311.2	97.5
CUC-DG-UC CD63-PE	1807.92	36158	339	142.8
CUC-DG-UC CD63-PE triton	1637.66	32753	323.5	112.5
qEV1-DG-UC unstained	1355.6	27112	340.3	90.8
qEV1-DG-UC CD63-PE	1275.56	25511	355	129.7
qEV1-DG-UC CD63-PE triton	1501.85	30037	315	112.1

Supplementary Table 2. Flow cytometry analysis of EVs isolated from pooled human serum, with the total events recorded and anti-CD63-PE median staining intensity with robust standard deviation (rSD) shown for unstained EVs and EVs labelled with anti-CD63-PE or anti-CD63-PE + 0.05% triton from each method alone or in combination.

Sample Name	Events/µL	Total events	Median R660-APC-H	rSD R660-APC-H
ExoQuick Plus unstained	3562.61	71252	223.5	85.5
ExoQuick Plus CD147-APC	1592.78	31852	328.3	198.1
ExoQuick Plus CD147-APC triton	1930.3	38606	243.4	145.3
UC unstained	4764.55	95290	219.3	86.6
UC CD147-APC	2621.75	52435	366.9	212.5
UC CD147-APC triton	2507.71	50154	245.6	143.8
CUC-UC unstained	5271.38	105427	222.4	86.9
CUC-UC CD147-APC	1799.56	35991	337.5	199.9
CUC-UC CD147-APC triton	1756.69	35133	237	140.8
qEV1 unstained	4245.25	84904	221.3	87.8
qEV1 CD147-APC	2417.33	48345	281.8	149.1
qEV1 CD147-APC triton	1983.75	39675	219.2	128.8
qEV2 unstained	3064.27	61285	223	86.9
qEV2 CD147-APC	2551.71	51034	294.7	165.7
qEV2 CD147-APC triton	2314.36	46287	229.1	134.8
DG-UC unstained	1866.1	37322	218.4	88.7
DG-UC CD147-APC	1534.21	30684	277.8	159.5
DG-UC CD147-APC triton	1681.95	33639	258.3	127.8
CUC-DG-UC unstained	2240.01	44800	221.7	88.8
CUC-DG-UC CD147-APC	1753.65	35073	276.6	154.5
CUC-DG-UC CD147-APC triton	1910.56	38211	251.9	130.9
qEV1-DG-UC unstained	1355.6	27112	244.9	84.2
qEV1-DG-UC CD147-APC	1475.95	29519	302.5	149.4
qEV1-DG-UC CD147-APC triton	1631.89	32637	252.2	126.9

Supplementary Table 3. Flow cytometry analysis of EVs isolated from pooled human serum, with the total events recorded and anti-CD147-APC median staining intensity with robust standard deviation (rSD) shown for unstained EVs and EVs labelled with anti-CD147-APC or anti-CD63-PE + 0.05% triton from each method alone or in combination.

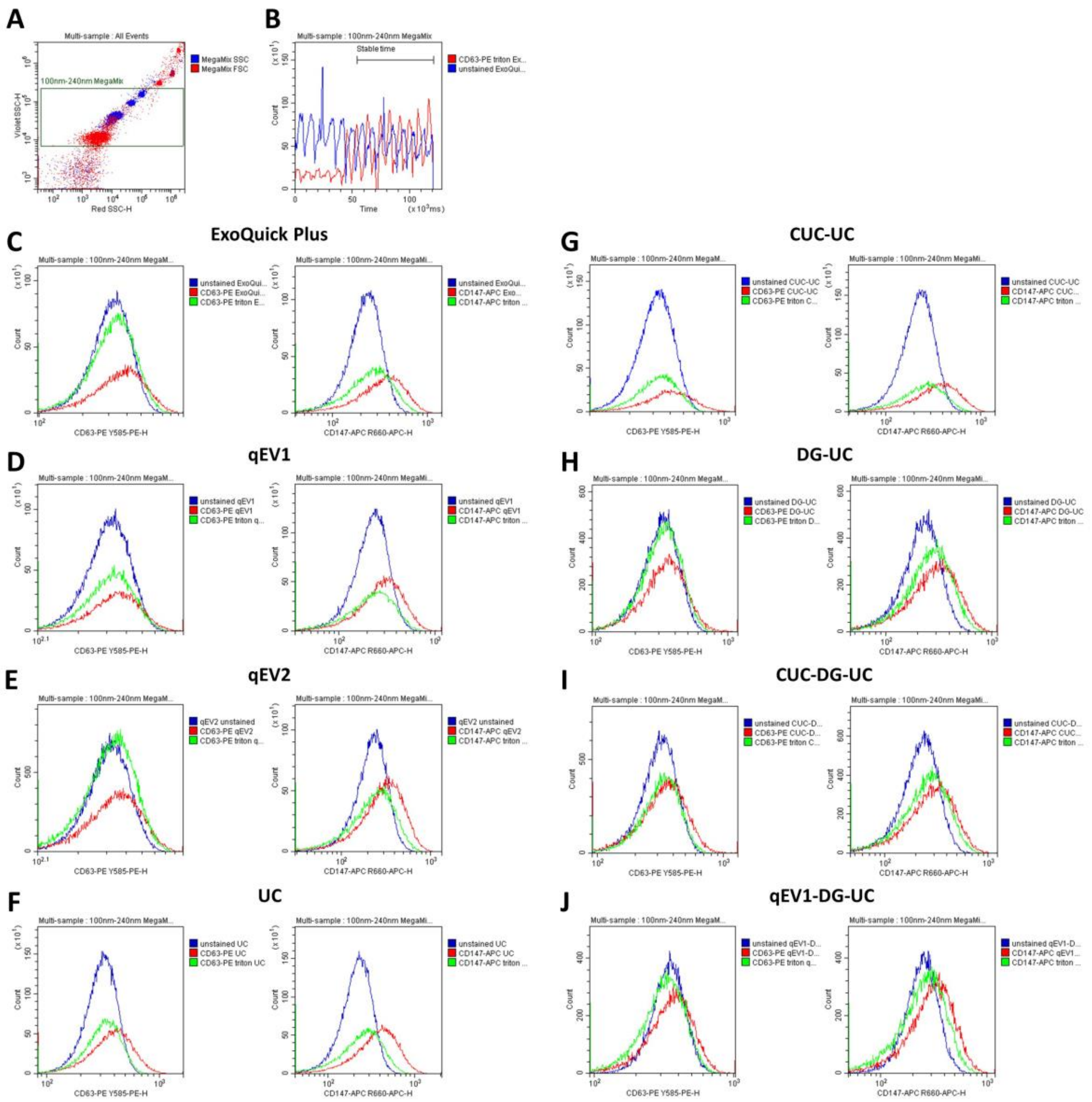

Supplementary Fig. 1. Flow cytometry analysis of EVs isolated from pooled human serum.

Construction of the microparticle region is based on (A) violet angle light scatter from 100 to 240nm polystyrene microspheres creating a ‘100nm-240nm Megamix gate’ and (B) a “stable time gate” based on a time histogram of the event counts in triton treated samples. (C-J) Histograms of EVs from each method alone or in combination, with unstained EVs (Blue), antibody labelled EVs (Red), or antibody labelled EVs + 0.05% triton (green). EVs were labelled with 0.05ul/100ul PBS anti-CD63-PE (H5C6, BD Bioscience (left) or 0.05ul/100ul PBS CD147-APC (MEM-M6/1, Thermo Scientific) (right) with/ without 0.05% triton for 30mins on ice in the dark.

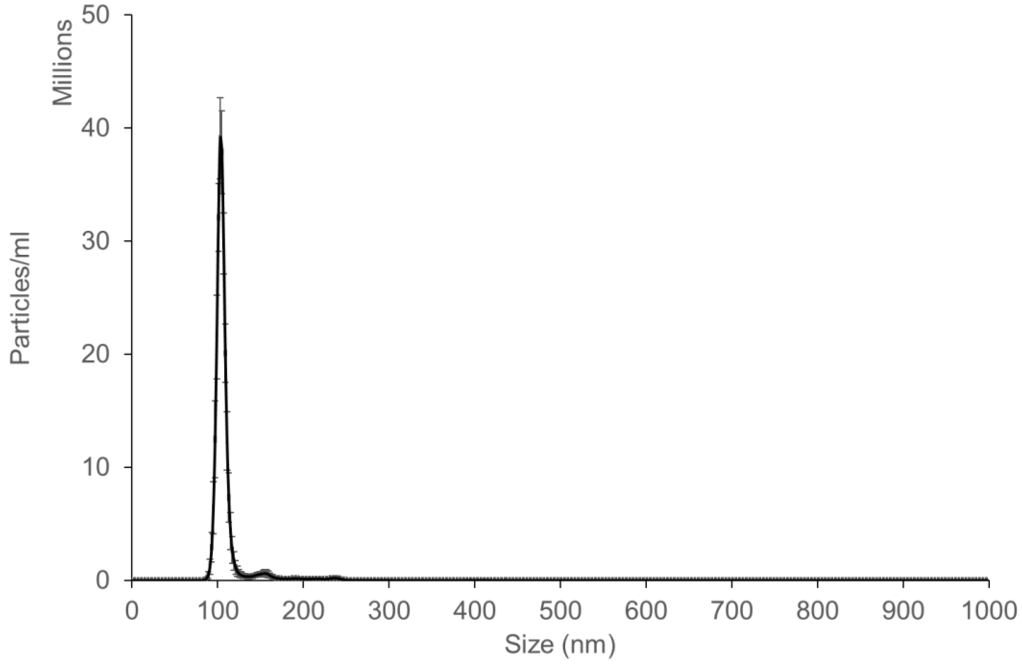

Supplementary figure 2. The NS300 Nanosight has been maintained under a service contract with Malvern Panalytical with monthly calibration verification using a 100nm nanosphere size standard (LTX3100A, Thermofisher Scientific), with the line graph being calculated from the mean of 5 1min videos.
